# Comparison of patients’ acceptance of cuff-based vs wearable 24-hour ambulatory blood pressure monitoring devices: A multi-method study

**DOI:** 10.1371/journal.pone.0336961

**Published:** 2025-11-17

**Authors:** Ariffin Kawaja, Aminath Shiwaza Moosa, Eric Kam Pui Lee, Ian Kwong Yun Phoon, Andrew Teck Wee Ang, Zi Ying Chang, Aileen Chelsea Ai’En Lim, Jonathan Yap, Weiting Huang, Ding Xuan Ng, Melvin Yuansheng Sng, Hao Yuan Loh, Chirk Jenn Ng

**Affiliations:** 1 SingHealth Polyclinics, Singapore, Singapore; 2 SingHealth-Duke NUS Family Medicine Academic Clinical Programme, Singapore, Singapore; 3 Jockey Club School of Public Health and Primary Care, The Chinese University of Hong Kong, HKSAR, China; 4 National Heart Centre Singapore, Singapore, Singapore; 5 Duke-NUS Medical School, Singapore, Singapore,; 6 Ministry of Health Holdings (Singapore) Private Limited, Singapore, Singapore; Warren Alpert Medical School of Brown University: Brown University Warren Alpert Medical School, UNITED STATES OF AMERICA

## Abstract

**Introduction:**

Recent hypertension guidelines recommend ambulatory blood pressure monitoring (ABPM) for accurate diagnosis and monitoring. However, patients’ experiences with cuff and wearable ABPM devices in primary care remain unclear. This study compared the acceptance of three devices (oscillometry cuff, tonometry wrist, and photoplethysmography chest devices) among patients with hypertension in primary care.

**Methods:**

A multi-method study was conducted. Thirty-five participants with hypertension were recruited from two public primary care clinics in Singapore. All participants used cuff-based and either wrist or chest wearable devices for 24 hours. Structured surveys and in-depth audio-recorded interviews were used to gather feedback on their views, experiences, and challenges using the devices. The interviews were thematically analysed, and the surveys were analysed using descriptive statistics.

**Results:**

All participants used the cuff (n = 35) device, while the wrist and chest devices were used by two-thirds (n = 22) and a third (n = 11) of the participants, respectively.

The device usability questionnaire found that most participants were satisfied with the chest device, which did not disrupt their daily activities. Conversely, cuff arm devices interfered with daily activities (48%) and sleep (26%), were cumbersome (32%), and caused embarrassment (26%). The wrist device was uncomfortable (33%) and painful (22%) for some participants.

The qualitative data were categorised into five themes: comfort, convenience, perceived accuracy, and impact on routine and sleep. Participants found the chest device more comfortable and convenient than the cuff and wrist devices. The cuff device was perceived as the most accurate due to its inflation-based BP measurement. All devices minimally affected routines and sleep, though participants expressed safety concerns about the cuff device, particularly while driving.

**Conclusion:**

While wearable ABPM devices offer increased comfort, convenience and reduced impact on patient’s daily activities, concerns regarding their accuracy must be addressed before the widespread adoption of these devices in routine clinical practice.

## Introduction

International hypertension guidelines recommend using ambulatory blood pressure monitoring (ABPM) devices for diagnosing and monitoring hypertension [1–4]. ABPM is most predictive of cardiovascular events [5] and prevents over-treatment in patients with whitecoat hypertension [6], resulting in cost savings. Additionally, ABPM implementation has demonstrated potential for healthcare cost reduction (3–14%) and decreased treatment days (10–23%) [7]. While some home BP devices [8] measure night-time BP, ABPM remains the reference standard to detect nocturnal hypertension, morning surge, dipping status, and 24-hour variability [9].

Despite these advantages, implementing ABPM in clinical practice remains challenging due to discomfort [10] and interference with sleep and daily activities [11], contributing to its lower acceptance compared to other BP measurement methods [12]. In the past decade, ABPM-related research has focused mainly on conventional cuff-based devices [13], which use oscillometry and are considered the gold standard.

However, the quest for more comfortable and convenient ABPM solutions has led to the emergence of wearable devices utilising technologies such as photoplethysmography (PPG) and tonometry [13,14]. These devices, using cloud-based software and AI [15] and integrated into user-friendly formats like rings, wristbands, smartphones [3], and patches [16], hold the promise of improved patient tolerance and potentially greater accessibility to ABPM. While current wearable devices may not yet possess an extensive clinical evidence base compared to traditional cuff-based devices, their development represents a significant step towards more patient-centric ABPM.

Most studies have focused on comparing these devices’ accuracy and clinical efficacy [17,18]. However, understanding the user experience of these devices is a crucial step, as greater comfort and convenience could ultimately lead to improved patient acceptance of ABPM, a vital factor in the effective implementation of ABPM service. Few studies have reported users’ acceptance of these devices [19,20], particularly in primary care settings where hypertension is prevalent. Furthermore, we anticipate that ongoing technological advancements and rigorous research will contribute to refining and validating these wearable technologies, potentially leading to clinically reliable alternatives to traditional ABPM.

In addition to the accuracy of the devices, understanding the experiences and acceptance of cuff and wearable devices are crucial for successfully implementing ABPM. Thus, our study aimed to explore the user experiences and acceptance of cuff and wearable devices for ABPM in a primary care setting. We compared a conventional cuff-based oscillometric device with two wearable devices: a ‘wrist device’ using tonometry technology and a ‘chest device’ using PPG technology. By identifying the users’ experience and acceptance of these devices, healthcare providers can strategise the implementation of ABPM to increase its uptake among patients with hypertension in primary care settings.

## Materials and methods

### Study design

This study used a multi-method design, comprising (i) a cross-sectional questionnaire survey examining user acceptance of the various ABPM devices and (ii) individual qualitative in-depth interviews (IDI) exploring their experiences and reasons for the acceptance of the devices, with reference to responses from the quantitative questionnaire. The concurrent method enabled the qualitative interview to expand on the closed-ended survey, providing deeper insights. The study is reported according to COREQ checklist (Appendix A in S1 File)

The survey and interviews were conducted after the participants completed the 24-hour ABPM using the cuff-based device and one of the two wearable devices for at least 24 hours. The reason for putting on two devices for each participant was to allow them to simultaneously experience both the wearable ABPM device and the gold-standard oscillometric ABPM device for real-time comparison [21]. A decision was made not to put on all three devices for each participant to reduce the research burden and technology-related fatigue [22].

**Fig 1:**
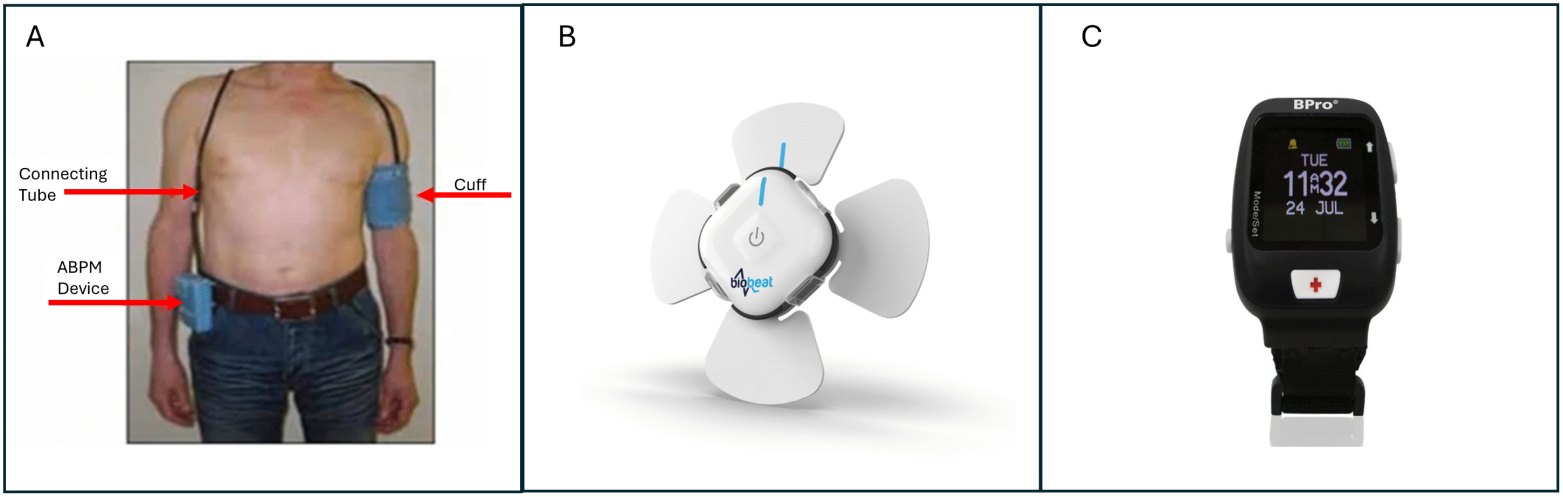
Ambulatory blood pressure monitoring devices. **A.** Spacelabs cuff monitor (Oscillometry); **B.** BioBeat chest wearable (Photoplethysmography); **C.** BPro watch wearable (Tonometry). The respective device companies have permitted the use of the images.

### Setting and participants

The study was conducted in two public primary care clinics in Singapore, with recruitment from March 2023 to January 2024. Participants were eligible if they were 21 years or older, had a diagnosis of hypertension confirmed by electronic medical records, and were referred for ABPM by their attending physician, irrespective of the indications. Exclusion criteria included individuals with cognitive impairments or physical disabilities that restricted the use of ABPM devices.

### Research team

The multidisciplinary research team comprised researchers with experiences in quantitative, qualitative and mixed-methods methodologies; they included: a senior male family physician (CJN), a female family physician (ASM), a male research fellow (AK), a male medical student (MYS), and a male data scientist (DXN).

### Recruitment of participants

Eligible participants referred for ABPM services were approached by study team members to gauge their interest in the study; those who wished to participate provided written informed consent before enrolling. The recruitment continued until data saturation was reached [23,24], when no new views, experiences, or challenges emerged from interviews and data analysis [25].

### Study instruments and data collection

#### Sociodemographic data collection form.

The questionnaire collected information on sociodemographic data, including age, gender, ethnicity, educational level, occupation, and medical information (Appendix B in S1 File). Participants completed this questionnaire at the time of recruitment.

#### Ambulatory blood pressure monitoring devices and their usage.

Three types of ABPM devices were investigated: [1] oscillometry cuff device (Spacelabs [26]), [2] tonometry wrist device (BPro [27]), and [3] photoplethysmography chest device (BioBeat [28]) (Fig 1). All three devices are approved for use by the Health Science Authority, Singapore (a local authority that regulates health products, including wearables) [29] and the Food and Drug Administration, USA [30–32]. The researcher received training from the respective vendors to ensure proper use and setup of these devices. Details about the specific ABPM devices can be found in Appendix C (S1 File)

After completing the sociodemographic data collection forms, two ABPM devices were applied to the participants: cuff-based and either wrist or chest devices. The wrist device was worn on the opposite wrist from the cuff device. The wearable device (wrist or chest) was selected based on its availability and compatibility with the participant’s mobile phone. Due to logistical reasons, the chest device was obtained later in the study, making it available only for participants recruited at that time. A one-point calibration was done as per manufacturer instructions, followed by a minimum of three BP readings for each participant using a clinically validated digital oscillometric cuff-arm device.

Participants wore the devices outside the clinic setting for 24 hours, recording BP at regular intervals (every 15 minutes for wearable and every 30 minutes for cuff-based) during both wake and sleep times. As indicated in previous studies, shorter intervals for the wearable devices were made possible because they were less intrusive [33,34].

Participants were unable to view their BP readings on the ABPM devices as the study team has opted to disable the function to reduce potential patient anxiety, which may influence their BP. The participants were aware when BP was taken on the cuff (cuff inflation/deflation) and wrist devices (a beep followed by a tick symbol on the wrist display); however, this was not the case for the chest device as BPs were measured discreetly without any physical, visual or auditory cues.

**Fig 2:**
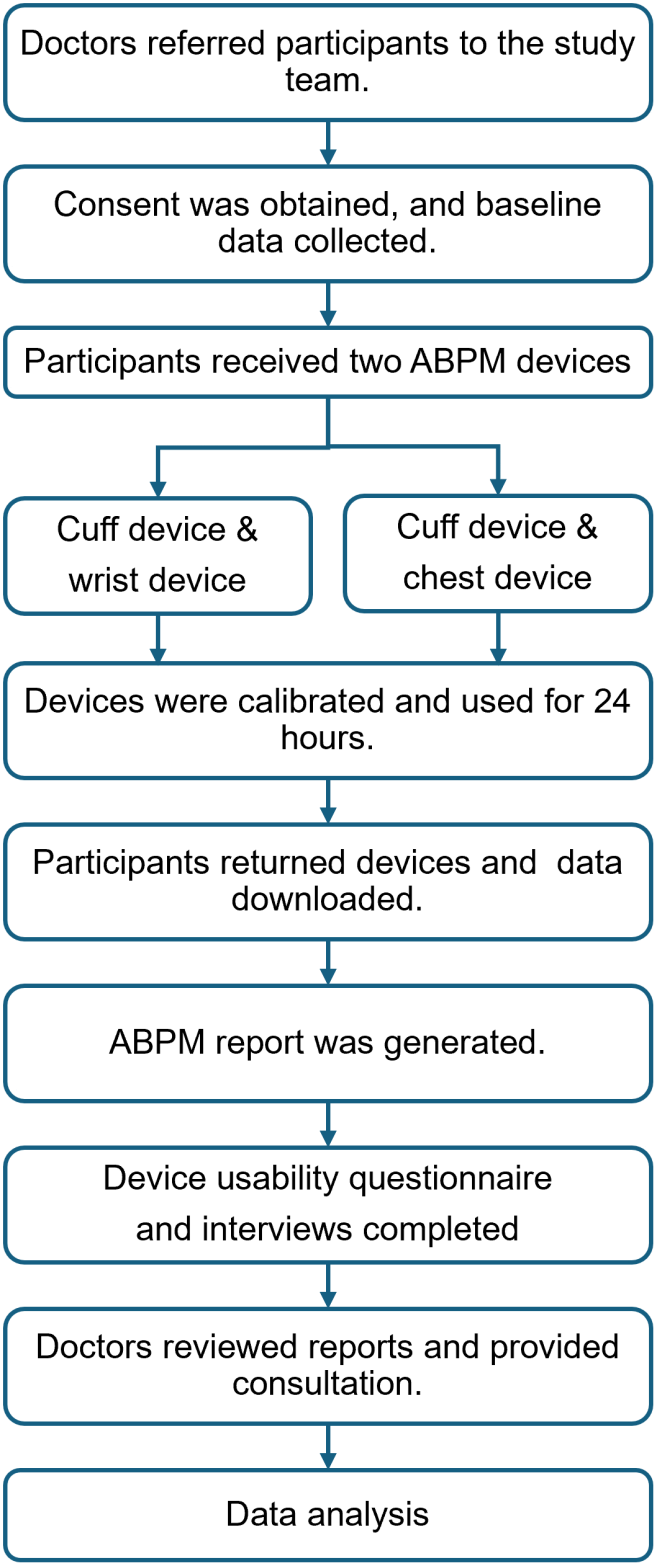
Flow diagram on study procedures and data collection.

After participants returned the devices the next day, data were downloaded directly from the devices (cuff and wrist device) or the cloud platform (chest device). Fig 2 illustrates the study procedure and data collection.

#### Device usability questionnaire completion.

Participants were asked to complete the device usability questionnaire when they returned with their devices. The eight-item questionnaire, developed based on the literature review, graded each item using a 5-point Likert scale from 1 (strongly disagree) to 5 (strongly agree). Each device has a separate usability questionnaire (Appendix D in S1 File).

#### Topic guide and interviews.

A semi-structured topic guide was developed based on the literature review and discussion. The questions explored the participants’ views, experiences and challenges in using the devices for ABPM (Appendix E in S1 File).

Trained qualitative researchers (CJN, AK and MYS) used the topic guide for the one-to-one interviews conducted on-site, in-person and in the participant’s preferred language (English or Mandarin). The interviewer was fluent in the language preferred by the participant, removing the need for a translator. The interviews lasted 30–60 minutes. No repeat interviews were conducted. Field notes were maintained during and after the interviews. All interviews were audio-recorded and securely stored on encrypted devices.

### Data analysis

#### Quantitative results.

Based on the baseline data collection, descriptive statistics were computed to describe the participants’ demographics. The usability for each device was analysed using proportions (%) of which scores ‘1’ and ‘2’ were grouped as ‘disagree’, ‘3’ as ‘neutral’, and ‘4’ and ‘5’ as ‘agree’.

#### Qualitative results.

All the recorded interviews were transcribed verbatim, checked independently, and analysed using a thematic approach.[35] Two researchers (ASM & AK) independently coded the first three interviews line-by-line to generate the initial codes (open coding), and the combined codes were reviewed by another researcher (CJN). Similar codes were grouped into categories and further rearranged into an initial coding frame (axial coding). The researchers analysed subsequent interviews independently, looking for emerging themes based on the coding frame and managed using NVivo (version 14) qualitative data management software. The researchers discussed the codes regularly, and any discrepancies were resolved by consensus. Representative quotes were selected to illustrate the participants’ experiences and acceptance of the ABPM devices. The coding tree is provided in Appendix F (S1 File)

### Reflexivity

As ASM and CJN were clinicians embedded within the primary care setting, we remained aware of how our professional roles and prior assumptions could shape the research process. We approached data collection and interpretation with a reflexive stance, acknowledging that our familiarity with management of hypertension and clinical workflows might have influenced how the interview questions were framed and the data were interpreted. Independent and iterative analysis, and regular team discussions between clinician and non-clinician researchers helped to surface and challenge these assumptions, ensuring that the participants’ perspectives remained central to our findings.

### Ethics considerations

The study was approved by the SingHealth Centralised Institutional Review Board (CIRB 2022/2517). All participants provided their written informed consent which was witnessed and documented in study file and participants case notes. All data were de-identified. The research was conducted according to the International Conference on Harmonisation Guidelines for Good Clinical Practice. Each participant was given a shopping voucher worth SGD$20 (USD$14.8) for their time and contributions.

### Patient and public involvement

Patient and public members reviewed the study protocol and provided feedback to revise the protocol before study initiation.

## Results

### Participant profile

A total of 35 patients participated in this study (Table 1). Participants declined to participate primarily due to lack of interest or time constraints. Most participants recruited were female (51.4%), of Chinese ethnicity (94.2%), had at least secondary education (85.7%), were employed (51.4%), and regularly measured their home BP (85.7%). All participants received the cuff arm ABPM, while the wrist and chest devices were used by two-thirds (n = 22) and a third (n = 11) of the participants, respectively. Almost half of the participants (48%) were referred by the physicians for ABPM for uncontrolled hypertension. Data from one wrist device and one chest device were unavailable; the former failed to capture data due to poor contact with the radial artery, while the latter was because the participant removed the device for work activities.

**Table 1:** Demographic profile of participants (n = 35).

Characteristics	Number	%	Mean ± SD (Range)
**Age**			62 ± 12.4 (32–80)
**Sex**			
Female	18	51.4	
Male	17	48.6	
**Ethnicity**			
Chinese	33	94.2	
Malay	1	2.9	
Indian	1	2.9	
**Education**			
No formal education	0	0	
Primary school	5	14.3	
Secondary school	11	31.4	
Diploma/Certificate	11	31.4	
Degree	5	14.3	
Postgraduate	2	5.7	
Others	1	2.9	
**Employment**			
Employed	18	51.4	
Unemployed/Retired	17	48.6	
**Devices worn by participants**			
Chest (BioBeats)	11	31.4	
Wrist (BPro)	22	62.9	
Cuff (Spacelabs)	35	100	
**Chronic medical conditions**			
Diabetes	9	25.7	
High Cholesterols	18	51.4	
Chronic kidney disease	2	5.7	
Heart disease	4	11.4	
Stroke	2	5.7	
Others	18	51.4	
**Duration with hypertension** (years)			6.6 ± 7.8 (0.1–30)
**Monitor blood pressure at home**			
Yes	30	85.7	
No	5	14.3	
**Indication for ABPM referral**			
Uncontrolled hypertension	15	48	
Suspected whitecoat uncontrolled hypertension*	9	26	
Fluctuating BP	8	23	
Newly diagnosed hypertension	2	6	
Suspected Hypertension	1	3	

* home BP controlled, but clinic BP uncontrolled in patients with hypertension.

### Usability survey findings

**Fig 3:**
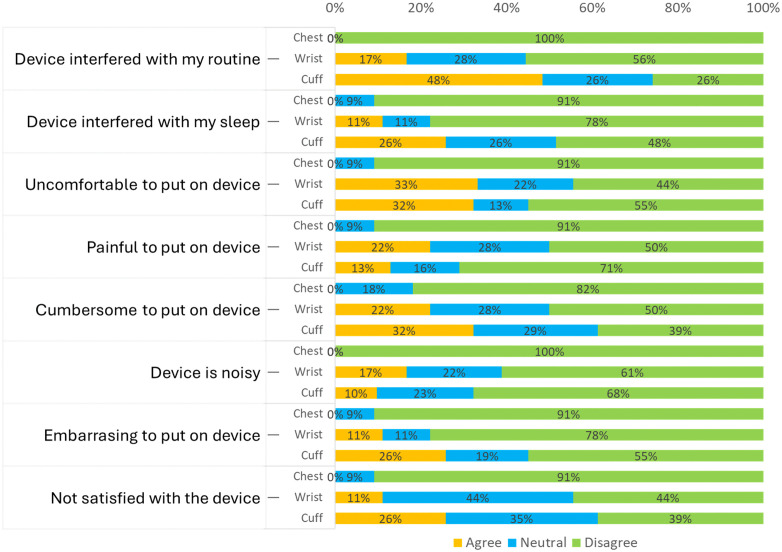
Usability questionnaire results across the three ABPM devices.

Most participants did not report discomfort, pain, or embarrassment from the chest device; there was no interference with their daily routines or sleep. In contrast, the cuff arm device impacted participants’ daily activities (48%) and sleep (26%); some found wearing it cumbersome (32%) and reported embarrassment (26%). Some participants found the wrist device uncomfortable (33%) or painful (22%). (Fig 3)

### Qualitative results

**Fig 4:**
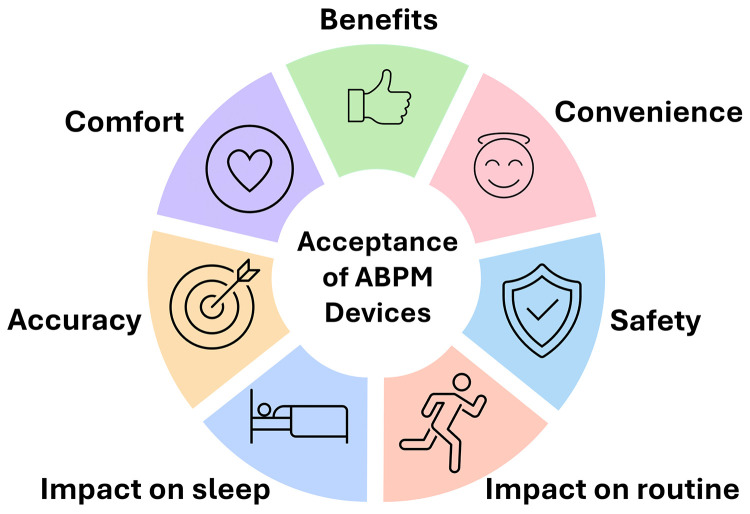
Themes on patients’ acceptance of ABPM devices.

The interviews yielded major themes on patients’ acceptance of ABPM devices: comfort, convenience, perception of accuracy, impact on routine, impact on sleep, benefits and safety (Fig 4)

#### Comfort.

Patients’ perspectives on comfort across the three devices differed. Some participants experienced discomfort or pain, rash and itch with the cuff and wrist devices. Some participants attributed the wrist device’s pain to the strap being tight or “bulky”.

*“But other than that, it’s the rashes. Because if I am in the aircon environment (it) is fine, but if I am out of aircon (airconditioned) environment, it starts to develop the rashes.”*

- P1 (Male, 57 years old, cuff & wrist device)

*“…after wearing it for twenty-four hours, the watch is really uncomfortable because of its size. It is very bulky, especially when it is rested on your wrist, and it really caused some pain.”*

*-* P12 (Female, 53 years old, cuff & wrist device)

A few participants shared the tightness felt while the cuff device was pumped, which resulted in patients experiencing pain, numbness, and a change in colour in their hands.

*“I think I feel more pain than numb. I can feel like it is very painful, but I don’t want to interfere with the readings, so I just endure. That’s where I see my hands all turn purple.”*

- P28 (Female, 33 years old, cuff & chest device)

Compared to the cuff and wrist devices, most participants using the chest device were pleased with the level of comfort the device provided. Only a few found the adhesive used for the chest device ‘sticky’.

#### Convenience.

While some participants perceived the cuff device as bulky and restrictive, drawing unnecessary attention, others appreciated its convenience due to easy removal for activities such as bathing.

“*I just walked and turned around, and there’s a chair, and it (the tube of the cuff device) got entangled. I feel a bit inconvenienced in that way.*”

- P1 (Male, 57 years old, cuff & wrist device)

“*For the cuff one, I think sometimes carrying the bulky thing, the wiring and the fact that it exposes the cuff pressure part. (That’s) when (other) people start asking (about the device).*”

– P16 (Female, 65 years old, cuff & wrist device)

Some who wore the wrist device were concerned that the wrist movements would displace the sensor.

“*So, I do not know how to check if the gadget is in the right position or not, especially after the base becomes loose.*”

- P3 (Male, 72 years old, cuff & wrist device)

Most participants found the chest device convenient, and several suggested it would be a suitable replacement for the cuff device. However, several participants faced problems during bathing as the device was not waterproof. A participant was concerned that the chest device requirement to use Bluetooth-enabled mobile phones and the Internet may limit its use for older adults.

*“I am also afraid, for older folks. In case of disconnection, whether they know what to do! Let’s say if it’s a bad (error) reading then some old folks know what to do next.”*

- P18 (Female, 68 years old, cuff & chest device)

#### Perception of accuracy.

Despite expressing more discomfort and inconvenience, participants perceived the cuff device as an accurate ABPM device as the device inflated and ‘physically’ measured the BP. On the other hand, the participants were concerned about the wrist device’s accuracy in capturing data due to wrist movements or potential sensor displacement.

*“I would recommend the cuff, despite this and that. No matter, you still take physical reading. The wrist one, you don’t know what kind of matrix they use to calculate the pressure. But the cuff physically pumps and takes the blood pressure. So, to me (cuff device) is more accurate.”*

- P1 (Male, 57 years old, cuff & wrist device)

One participant doubted the overall accuracy of ABPM as it did not reflect his usual lifestyle and routine.

“*First thing, it’s not comfortable. Second thing, I cannot sleep. Third thing, I cannot shower. It’s not my usual lifestyle. Your data, I do not know; it is not accurate.”*

- P31 (Male, 62 years old, cuff & chest device)

#### Impact on routine.

The impact on the participants’ routines during ABPM was minimal except during physical exercises. Most participants avoided physical activities when they put on the cuff device as they felt the data capture would be affected. Some participants shared that they tended to stay home instead of outdoors when using any device.

“*Because I’m not working and I am at home most of the time, so it is okay. But, if I am outside working, I can guarantee it will not capture the right readings because when you’re at work, you rush here and there.*”

- P13 (Female, 48 years old, cuff & wrist device)

One participant shared that the pumping of the cuff device could disrupt their work activities, such as meetings.

*“…sometimes when I am in the office and it started pumping, it caused a little bit of disruption to my daily life, especially like when I was doing some discussion; initially I was very conscious and said, “Wait, I need to pump.” But after that, it’s like I cannot keep stopping the meeting just for it. So, I carry on.”*

- P27 (Female, 33 years old, cuff & chest device)

The cuff device was found to be the least disruptive for bathing because it could easily be removed and put on again. Similarly, most did not find this an issue with the wrist device because the splash cover protected it from getting wet. For the chest device, some participants chose not to shower when they put it on.

“*For the cuff, I took it out and timed myself every twenty minutes. I then put it back on so that I don’t miss the reading.*”

- P19 (Female, 35 years old, cuff & chest device)

*“You guys gave me the plastic cover previously. I put it on while taking a shower, and it is no problem. The plastic is very good - water didn’t seep in.”*

- P12 (Female, 53 years old, cuff & wrist device)

*“For the chest device, I needed to be extra careful because I was told it is not waterproof.”*

- P27 (Female, 33 years old, cuff & chest device)

#### Impact on sleep.

Most participants’ sleep was not affected by the three ABPM devices. Those affected reported they were light sleepers, and their sleep was mainly affected by the squeezing action of the cuff during measurement. Although they could hear a beeping sound from the wrist device, their sleep was unaffected; similarly, the chest device did not affect their sleep.

*“If you were to compare the cuff device to the normal desktop device, the pumping sound would not be as loud, so it is still bearable. This thing is on the wrist because when it does not capture the signal, after 20 seconds, it will run again until it capture the signal and the tick sign comes on, then the signal is correct. At the initial 5-10 minutes (of trying to sleep), I can hear (the sound from wrist device), but after that I knock off.”*

- P1 (Male, 57 years old, cuff & wrist device)

*“I was not able to go to sleep because the moment I wanted to sleep, that is when it (cuff device) started to take measurements. The cuff started to tighten, and I started to wake up.”*

- P27 (Female, 33 years old, cuff & chest device)

#### Benefits.

Some participants benefited from the ABPM by having a clearer picture of their BP pattern; they felt the 24-hour report could identify possible triggers or periods of uncontrolled BP. Doctors could use the generated report to adjust their medications.

*“I will still recommend, despite all these discomforts; our health is still a priority. If there is a device that can collect our data over a period of time, let us say 24 hours or 48 hours, then put it into the system for a diagnosis, it will be a clearer picture of what went wrong and how we are going to spot it.”*

- P1 (Male, 57 years old, cuff & wrist device)

*“For instance, he changed my medication today. He (the doctor) said: “Your blood pressure is still very high” after tracking with that device.”*

- P2 (Male, 72 years old, cuff & wrist device)

#### Safety.

The participants shared concerns regarding the safety of the cuff device, particularly during driving. They also reported that the tube of the cuff device could get entangled. Some were also concerned about hygiene, as different users might use the same arm cuff.

*“Another thing is, it (cuff device) interferes with the driving a lot. Driving is quite dangerous. Especially when you drive, and it starts to pump; it affects your ability to hold on to the steering wheel.”*

- P6 (Male, 44 years old, cuff & wrist device)

*“The only concern is the tubing (on the cuff device); it may pose a safety hazard, and sometimes it may hook onto the chair.”*

- P7 (Male, 72 years old, cuff & wrist device)

## Discussion

This study shed light on the often-overlooked aspect of patients’ acceptance and experience using an ABPM device. The cuff device was the most familiar and perceived as the most accurate, but it caused discomfort and posed safety concerns while driving. The wrist device was bulkier, leading to skin irritation and concerns about positional accuracy. The chest device was the most comfortable but lacked waterproofing and unclear measurement methods. Despite these differences, the devices did not disrupt sleep, and the participants recognised the value of ABPM for continuous blood pressure monitoring.

Previous studies have indicated that wearable devices were more comfortable than traditional cuff-based devices, as they did not require repeated inflations, which can be painful and disturbing [36,37]. In addition to the discomfort from the cuff device, our study revealed discomfort associated with the wrist device, attributed to its size and the tightness of the strap needed to secure the sensor. Future device design should minimise the pressure on the wrist while ensuring sufficient contact for accurate data capture [38]. Based on our findings, the chest device caused minimal discomfort and disruption to daily life, likely increasing users’ acceptance and uptake of ABPM devices in clinical settings.

There was a safety concern related to the cuffed ABPM device, specifically the risk of the connecting tube becoming entangled in furniture, such as chairs, and causing hindrance to steer while driving. These experiences underscore potential real-world safety challenges beyond the discomfort of cuff inflation. While existing guidelines [39] on ABPM advise against driving due to the distraction caused by cuff inflation, our findings suggest that the physical presence of the tubing also poses a practical safety risk that warrants consideration. Reports of steering hindrance reinforce the need for caution, demonstrating that the device’s physical components can interfere with safe vehicle operation. These findings emphasise the need for patient selection, patient education on potential entanglement hazards, and further design considerations in ABPM devices to minimise such risks in daily use.

Our study highlighted a notable inconvenience regarding daily activities, particularly bathing, with wearable devices, causing some participants to forgo bathing during the monitoring period. While some wrist devices offer limited water resistance against splashes, submersion remains problematic [40]. Similarly, chest devices, with their electronic components and skin adhesives, are susceptible to water damage [41]. The lack of waterproofing across wearable ABPM devices presents a significant inconvenience for bathing and swimming, potentially disrupting daily routines. Furthermore, this limitation could also hinder religious practices, such as Islam, which require regular washing. Consequently, this inconvenience may impede the broader adoption of wearable ABPM, as the need for device removal or water avoidance can disrupt users’ lifestyles and the continuous nature of monitoring, thereby impacting the compliance and overall effectiveness of these technologies.

A third (32%) of the participants reported that the cuffed ABPM device was cumbersome compared to the cuffless wearables; this is due to the bulky monitoring unit and inflatable cuff, as well as the connecting tube which restricts clothing choice and patient mobility [42]. Furthermore, the cuffed device disrupts patient’s daily activities due to its regular inflation and sound throughout the day. In contrast, the wrist and chest devices are typically smaller and operate discreetly, offering convenience and flexibility [42,43]. In addition, the cuffed device may be associated with stigma for some patients. Existing literature confirms that social awkwardness and stigma associated with wearing obvious medical instruments limits its acceptance [44]. Conversely, cuffless wearable devices minimize embarrassment, and more likely to improve user experience and, therefore, enhance compliance. [42]

Despite recognising the potential benefits of wearable blood pressure monitoring, the American Heart Association [45] and European Society of Hypertension [2] have cautioned against wearable device use for clinical management, citing accuracy issues and uncertain applicability. Participants perceived wearable devices as less accurate than cuff-based monitors due to concerns about positional changes affecting wrist device readings and uncertain data capture and analysis processes. The literature highlights the difficulty in achieving comparable accuracy with wearable technologies and emphasises the need for transparency to build clinical confidence [1,46]. The unclear measurement process remains a barrier to adoption [47]. Future research should mandate complete transparency in data acquisition, processing algorithms, and validation methodologies while improving wearable ABPM technologies’ accuracy. This would enhance trust, demystify concerns, foster trust among clinicians and patients, and support broader clinical integration of these devices.

Reassuringly, none of the ABPM devices, including the cuffed device, significantly disrupted the participants’ sleep. This finding contrasts with existing literature that indicates the periodic inflation of the cuff during nocturnal monitoring can cause sleep disturbance, leading to fragmented sleep and reduced sleep quality [48]. In addition, sleep disruption may affect night-time BP readings, which affects the accuracy of ABPM and increases the risk of misdiagnosis and inappropriate management [49]. However, our findings align with other studies suggesting that advancements in device design, such as quieter and faster inflation cycles, may have mitigated these issues [50,51]. While our findings are reassuring, clinicians should still inquire about sleep quality during ABPM to ensure accurate nocturnal BP assessment. Further research with objective sleep measures is warranted to definitively evaluate the impact of modern ABPM devices on sleep and the reliability of nocturnal BP data.

### Strength and limitations

To the authors’ knowledge, this is the first study exploring the patients’ experiences of receiving various ABPM devices among an Asian population in a primary care setting. Using cuff-based and wearable devices simultaneously enabled patients to directly compare their experiences with each device in real time. Concurrent quantitative and qualitative interviews enabled triangulation of findings from both methods, offering a comprehensive understanding of device acceptability.

There are several limitations to this study. Although international guidelines recommend conducting ABPM on workdays [1], many patients took leave or worked from home to minimise their movements, which may affect their BP readings. As a result, their experiences with routine activities may not have been fully captured.

Another limitation is that the devices selected for this study cannot represent the other devices in the market. There could be usability differences between the chosen cuff, wrist, and chest devices. The technologies (oscillometry, tonometry, and photoplethysmography (PPG)) adopted by different manufacturers would be different and may provide different experiences. However, this study provides insight into the attributes of ABPM devices when implementing ABPM in clinical practice.

The Chinese ethnic group is also over-represented in this study’s sample population. Experiences by the other ethnic groups should ideally be captured. For example, cultural and religious practices would require the removal of the wrist device to wash their hands and arms before performing their prayers. These practices are nuanced for such minority groups such as the Malay and Indian Muslims and would explain why specific devices are more acceptable than others. Future studies should include variations in ethnicity to ensure the research is inclusive and considerate.

We did not explore the cost of the devices, which is likely an important factor influencing patients’ acceptance and the feasibility of implementing them in primary care. A cost-effectiveness analysis is needed to compare the use of ABPM with referrals to hospitals, which is the current practice locally.

### Conclusion

Implementing ABPM as part of clinical care must consider patients’ acceptance of the device attributes, such as portability, comfort, disruption of daily life, and perceived accuracy. Wearable ABPM devices can potentially increase accessibility to ABPM; however, their accuracy remains a concern and must be addressed before they can be used to support healthcare providers more widely in clinical decision-making and benefit patients with hypertension.

## Supporting information

S1 File Appendix A-E. (DOCX)

## Abbreviations

ABPM: Ambulatory blood pressure monitoring
BP: Blood pressure.
