# Supplementary material for: Comparison of patients’ acceptance of cuff-based vs wearable 24-hour ambulatory blood pressure monitoring devices: A multi-method study
Source: PLoS One. 2025 Nov 17;20(11):e0336961. doi: 10.1371/journal.pone.0336961 (PMC12622775; doi:10.1371/journal.pone.0336961)
Supplement: S1 File — (DOCX) [file pone.0336961.s001.docx]

**Appendix A**

**Title**: Comparison of patients' acceptance of cuff-based vs wearable 24-hour ambulatory blood pressure monitoring devices: a multi-method study.

**Consolidated criteria for reporting qualitative studies (COREQ): 32-item checklist**

Developed from:

Tong A, Sainsbury P, Craig J. Consolidated criteria for reporting qualitative research (COREQ): a 32-item checklist for interviews and focus groups. *International Journal for Quality in Health Care*. 2007. Volume 19, Number 6: pp. 349 – 357

| **No. Item** | **Guide questions/description** | **Reported on Page #** |
| --- | --- | --- |
| **Domain 1: Research team and reﬂexivity** |  |  |
| *Personal Characteristics* |  |  |
| 1. Interviewer/facilitator | Which author/s conducted the interview or focus group? | Page 10 |
| 2. Credentials | What were the researcher’s credentials? E.g. PhD, MD | Page 7 |
| 3. Occupation | What was their occupation at the time of the study? | Page 7 |
| 4. Gender | Was the researcher male or female? | Page 7 |
| 5. Experience and training | What experience or training did the researcher have? | Page 7 |
| *Relationship with participants* |  |  |
| 6. Relationship established | Was a relationship established prior to study commencement? | Page 8-9  . |
| 7. Participant knowledge of the interviewer | What did the participants know about the researcher? e.g. personal goals, reasons for doing the research | Page 9-10 |
| 8. Interviewer characteristics | What characteristics were reported about the interviewer/facilitator? e.g. Bias, assumptions, reasons and interests in the research topic | Page 11 |

| **Domain 2: study design** |  |  |
| --- | --- | --- |
| *Theoretical framework* |  |  |
| 9. Methodological orientation and Theory | What methodological orientation was stated to underpin the study? e.g. grounded theory, discourse analysis, ethnography, phenomenology, content analysis | Page 6 |
| *Participant selection* |  |  |
| 10. Sampling | How were participants selected? e.g. purposive, convenience, consecutive, snowball | Page 6 |
| 11. Method of approach | How were participants approached? e.g. face-to-face, telephone, mail, email | Page 6 |
| 12. Sample size | How many participants were in the study? | Page 6 |
| 13. Non-participation | How many people refused to participate or dropped out? Reasons? | Page 12 |
| *Setting* |  |  |
| 14. Setting of data collection | Where was the data collected? e.g. home, clinic, workplace | Page 10 |
| 15. Presence of non-participants | Was anyone else present besides the participants and researchers? | Page 10 |
| 16. Description of sample | What are the important characteristics of the sample? e.g. demographic data, date | Page 12 |
| *Data collection* |  |  |
| 17. Interview guide | Were questions, prompts, guides provided by the authors? Was it pilot tested? | Page 10 & Appendix E |
| 18. Repeat interviews | Were repeat interviews carried out? If yes, how many? | Page 10 |
| 19. Audio/visual recording | Did the research use audio or visual recording to collect the data? | Page 10 |
| 20. Field notes | Were ﬁeld notes made during and/or after the interview or focus group? | Page 10 |
| 21. Duration | What was the duration of the interviews or focus group? | Page 10 |
| 22. Data saturation | Was data saturation discussed? | Page 7 |
| 23. Transcripts returned | Were transcripts returned to participants for comment and/or correction? | Page 6 |
| **Domain 3: analysis and ﬁndings** |  |  |
| *Data analysis* |  |  |
| 24. Number of data coders | How many data coders coded the data? | Page 10 |
| 25. Description of the coding tree | Did authors provide a description of the coding tree? | Page 10 & Appendix F |
| 26. Derivation of themes | Were themes identiﬁed in advance or derived from the data? | Page 10 |
| 27. Software | What software, if applicable, was used to manage the data? | Page 10 |
| 28. Participant checking | Did participants provide feedback on the ﬁndings? | No |
| *Reporting* |  |  |
| 29. Quotations presented | Were participant quotations presented to illustrate the themes/ﬁndings? Was each quotation identiﬁed? e.g. participant number | Page 8-13 |
| 30. Data and ﬁndings consistent | Was there consistency between the data presented and the ﬁndings? | Page 14-24 |
| 31. Clarity of major themes | Were major themes clearly presented in the ﬁndings? | Page 14-24 |
| 32. Clarity of minor themes | Is there a description of diverse cases or discussion of minor themes? | Page 14-24 |

**Appendix B**

**EmPaTHy-ABPM Project – Collection Form for Patient**

Assessing the feasibility and acceptability of using an ambulatory blood pressure monitoring wearable device to diagnose and monitor hypertension in primary care.

**Section A: Introduction**

The purpose of this Data Collection Form is to gather information about your particulars and clinical data. The information will be kept confidential and will be used strictly for research purposes only. This will help the researchers to understand your background when interpreting the results.

Please tick the checkboxes or fill in the blanks where appropriate.

**Section B: Your particulars**

| B1. | Age | | | | Years Old | | |
| --- | --- | --- | --- | --- | --- | --- | --- |
|  | | |  |  |  |  |  |
|  | |  | |  | |  |  |
| B2. | | Gender: | | - Female - Male | |  |  |
|  | |  | |  | |  |  |
| B3. | | Ethnicity: | | - Chinese - Malay - Indian - Others. Please specify: | |  |  |
|  | |  | |  | |  |  |

| B3. | | Are you currently working? | | - Yes, Please specify: | |  |
| --- | --- | --- | --- | --- | --- | --- |
|  | |  | - No | |  |  |
|  | |  |  |  |  |  |
|  |  |  |  |  |  |  |
|  |  |  |  |  |  |  |

| B6. | Highest Education Level | - No Formal Education - Primary School - Secondary School - Diploma/Certificate - Degree - Postgraduate - Others. Please specify: |  |
| --- | --- | --- | --- |

**Section C: Medical History**

| C1. | Chronic Medical Conditions   - Diabetes - High Cholesterol - Chronic Kidney Disease - Heart Disease - Stroke - Others |  |
| --- | --- | --- |

| C2. | How long have you had hypertension? | Years |
| --- | --- | --- |

| C3. | Do you monitor your own blood pressure at home? | - Yes - No |
| --- | --- | --- |

| C4. | Have you ever used any watches or patches  for continuous blood pressure monitoring? | - Yes, please specify:   _______________________   - No |
| --- | --- | --- |

------------------------------------------------ OFFICIAL USE ONLY -----------------------------------------------

| *To be filled in by Study Team* | |  |  |
| --- | --- | --- | --- |
|  | |  | Study site: |
| Participant Code: |  |  | - Sengkang |
| Date of Interview: |  |  | - Eunos |
| Start time: |  |  |  |
| End time: |  |  |  |
| ABPM device given |  |  |  |
| Reason for referral by doctor  to join study |  |  |  |
| Medication List: | ______________________________ |  |  |

**Appendix C**

Details of the ABPM devices used for the study.

**Oscillometry cuff device:** The oscillometry cuff consists of an inflatable cuff worn on the upper arm, connected with a tube to a portable lightweight recording device (Figure 1A). The cuff size was determined by measuring the upper arm circumference and selecting a cuff size according to the manufacturer's specifications. The cuff inflated to capture BP every 30 minutes throughout the 24 hours. Participants were allowed to remove the device during showering.

**Tonometry wrist device:** This cuffless wrist tonometry device resembles a large watch and includes a wrist strap fitted with a display screen on one side and a pressure module (pressure sensor with sensor plunger) on the other side; the sensor module uses tonometry technology and detects changes in the pulse pressure variability (at the radial artery) (Figure 1C) The wrist strap is adjustable for participant comfort and applanation of the radial artery to pick up the best signal. The BP recordings are captured every 15 minutes and transferred via Bluetooth interface to a mobile application. The data is then uploaded to a secure cloud platform via the Internet. The device is non-waterproof, and participants were provided with a plastic cover for use during a shower.

**Photoplethysmography chest device**: The device uses an optical technique to detect blood volume changes by analysing light absorption or reflection from the microvascular tissue of the skin. It includes a sensor capsule mounted onto a chest patch sticker connected to a mobile application, which captures the BP recordings every 15 minutes via Bluetooth interface; the captured data is uploaded to a secure cloud platform via the Internet (Figure 1B). It is a single use device and can be discarded after its use. As the device is non-waterproof, the participants were instructed to keep the chest patch dry and, if needed, to clean themselves with a wet cloth.

**Appendix D**

***Questionnaire for Patient Participants (Spacelab Blood Pressure Cuff Device)***

This questionnaire hopes to find out your experience in using the cuff blood pressure monitoring device. Please state to what extent you agree with each of the statements below from 1 'Strongly Disagree' to 5 'Strongly Agree' by circling the number that best reflects your experience

**Strongly Disagree** **Strongly Agree**

| 1 | 2 | 3 | 4 | 5 |
| --- | --- | --- | --- | --- |

1. I found the cuff device interfered with my daily

Activities most of the time.

| 1 | 2 | 3 | 4 | 5 |
| --- | --- | --- | --- | --- |

1. Wearing the cuff device interfered with my sleep.

| 1 | 2 | 3 | 4 | 5 |
| --- | --- | --- | --- | --- |

1. I found it uncomfortable to wear the cuff device.

| 1 | 2 | 3 | 4 | 5 |
| --- | --- | --- | --- | --- |

1. I found it painful to wear the cuff device.

| 1 | 2 | 3 | 4 | 5 |
| --- | --- | --- | --- | --- |

1. I found it cumbersome to wear the cuff device.

| 1 | 2 | 3 | 4 | 5 |
| --- | --- | --- | --- | --- |

1. I found the cuff device noisy.

| 1 | 2 | 3 | 4 | 5 |
| --- | --- | --- | --- | --- |

1. I found it embarrassing to wear the cuff device.

| 1 | 2 | 3 | 4 | 5 |
| --- | --- | --- | --- | --- |
| Not  satisfactory at all | Not  satisfactory | Neutral | Satisfactory | Very satisfactory |

1. Overall, how was your experience wearing the cuff device?

***Questionnaire for Patient Participants (BPro Blood Pressure Watch Device)***

This questionnaire hopes to find out your experience in using the watch blood pressure monitoring device. Please state to what extent you agree with each of the statements below from 1 'Strongly Disagree' to 5 'Strongly Agree' by circling the number that best reflects your experience

**Strongly Disagree** **Strongly Agree**

| 1 | 2 | 3 | 4 | 5 |
| --- | --- | --- | --- | --- |

1. I found the watch device interfered with my daily

Activities most of the time.

| 1 | 2 | 3 | 4 | 5 |
| --- | --- | --- | --- | --- |

1. Wearing the watch device interfered with my sleep.

| 1 | 2 | 3 | 4 | 5 |
| --- | --- | --- | --- | --- |

1. I found it uncomfortable to wear the watch device.

| 1 | 2 | 3 | 4 | 5 |
| --- | --- | --- | --- | --- |

1. I found it painful to wear the watch device.

| 1 | 2 | 3 | 4 | 5 |
| --- | --- | --- | --- | --- |

1. I found it cumbersome to wear the watch device.

| 1 | 2 | 3 | 4 | 5 |
| --- | --- | --- | --- | --- |

1. I found the watch device noisy.

| 1 | 2 | 3 | 4 | 5 |
| --- | --- | --- | --- | --- |

1. I found it embarrassing to wear the watch device.

| 1 | 2 | 3 | 4 | 5 |
| --- | --- | --- | --- | --- |
| Not  satisfactory at all | Not  satisfactory | Neutral | Satisfactory | Very satisfactory |

1. Overall, how was your experience wearing the watch device?

***Questionnaire for Patient Participants (BPro Blood Pressure Chest Device)***

This questionnaire hopes to find out your experience in using the chest blood pressure monitoring device. Please state to what extent you agree with each of the statements below from 1 'Strongly Disagree' to 5 'Strongly Agree' by circling the number that best reflects your experience

**Strongly Disagree** **Strongly Agree**

| 1 | 2 | 3 | 4 | 5 |
| --- | --- | --- | --- | --- |

1. I found the chest device interfered with my daily

Activities most of the time.

| 1 | 2 | 3 | 4 | 5 |
| --- | --- | --- | --- | --- |

1. Wearing the chest device interfered with my sleep.

| 1 | 2 | 3 | 4 | 5 |
| --- | --- | --- | --- | --- |

1. I found it uncomfortable to wear the chest device.

| 1 | 2 | 3 | 4 | 5 |
| --- | --- | --- | --- | --- |

1. I found it painful to wear the chest device.

| 1 | 2 | 3 | 4 | 5 |
| --- | --- | --- | --- | --- |

1. I found it cumbersome to wear the chest device.

| 1 | 2 | 3 | 4 | 5 |
| --- | --- | --- | --- | --- |

1. I found the chest device noisy.

| 1 | 2 | 3 | 4 | 5 |
| --- | --- | --- | --- | --- |

1. I found it embarrassing to wear the chest device.

| 1 | 2 | 3 | 4 | 5 |
| --- | --- | --- | --- | --- |
| Not  satisfactory at all | Not  satisfactory | Neutral | Satisfactory | Very satisfactory |

1. Overall, how was your experience wearing the chest device?

**Appendix E**

EmPaTHy-ABPM Project – Topic guide (Patient)

**Title**: Assessing the feasibility and acceptability of using an ambulatory blood pressure monitoring wearable device to diagnose and monitor hypertension in primary care: A multi-method study

**Preamble:**

- Ice-breaking session (introduce and get to know participants)
- Explain the purpose of the session (to find out your experience using the ABPM devices)
- Give time for the participant to go through the Participant Information Sheet (if not read yet)
- Interested in personal views and opinions
- No right or wrong answers
- Permission to refuse to answer questions
- Views will be kept confidential
- Obtain permission for audiotaping.
- Explain the confidentiality of recordings and data.
- Give opportunity for the participant to ask questions
- Obtain written consent
- Complete data collection form
- Any questions before we start?

**Opening questions**:

1. How would you describe your **overall experience** after using the ABPM study device?

Probe:

- Spacelabs BP cuff
- BPro watch/BioBeats chest device

1. How did the devices **affect your activities** throughout the **entire day**?

Probe: (For both devices)

- At Home?
- At Work?
- Driving?
- Showering?
- At other times?

1. How **comfortable** were you wearing the device(s)?

- Did you experience any **pain** when wearing the devices?
- Did you experience any discomfort while wearing the devices?

Probes:

- - Skin irritation e.g rash, itch
  - Bruising
  - Numbness
  - Any other experience?
- Which device was more comfortable? Why?

1. Did the study devices interfere with your **sleep**?

- Why or why not?
- If your sleep was disturbed, did the device..
  - Stop you from falling asleep
  - Wake you up after falling asleep
  - Disturb your sleep enough for you to remove it?
- Which device was more interruptive to your sleep?

1. What do you think about the devices?

Probes:

- Did the **noise** of the ABPM Blood pressure cuff disturb you? (at Work? Driving? At Home? At other times?)
  - Did it disturb you enough to remove it?
  - Did the noise of the pump disturb others?
- Did you find the device **heavy**?
- Did you find the monitor **embarrassing** to wear?
  - Why or why not?
  - Which device was more embarrassing?

1. Did you have to **remove** the device and **put it** **back on**? Why?

Probe: How easy or difficult was it for you to:

- Put on:
  - device(s)
  - plastic cuff on the device? (For bathing)
- Removing the device/cuff

1. Did you encounter any **error messages** on the devices?

- If you did, how did you feel when you saw the error message?
- What did you do afterwards?

1. Can you tell me **why you were advised** to undergo **ABPM**?

Probe:

- How did you feel having your blood pressure constantly monitored?
- Would you rather have your BP readings shown on the device on the spot when it is recorded? Why or why not?

1. For people who needs to monitor their BP for 24 hours, would you **recommend** them to use this ABPM device?

- Why or why not?
- Which devices would you recommend?

1. What do you think about the **activity log**?

- Was it easy for you to record your activities throughout the day?
- Do you think the 30-minute interval for activity log recording was easy to adhere to? Why or why not?

**Closing questions**:

1. Do you have any other comments about your experience with the ABPM wearable devices?

**Post-Interview**

- Indicate this is the end of the interview
- Thank the participant for their time and effort
- Hand the vouchers to the participant and ask them to sign the acknowledgment
